# Preliminary result of combined treatment with scanning carbon-ion radiotherapy and image-guided brachytherapy for locally advanced cervical adenocarcinoma

**DOI:** 10.1093/jrr/rrae043

**Published:** 2024-06-06

**Authors:** Keisuke Tsuchida, Daisaku Yoshida, Satoshi Shima, Terufumi Kusunoki, Yoshiki Takayama, Hiroaki Koge, Kio Kano, Yosuke Takakusagi, Nobutaka Mizoguchi, Tadashi Kamada, Yohsuke Kusano, Hisamori Kato, Hiroyuki Katoh

**Affiliations:** Department of Radiation Oncology, Kanagawa Cancer Center, 2-3-2, Nakao, Asahi-ku, Yokohama, Kanagawa 241-8515, Japan; Department of Radiation Oncology, Kanagawa Cancer Center, 2-3-2, Nakao, Asahi-ku, Yokohama, Kanagawa 241-8515, Japan; Department of Radiation Oncology, Kanagawa Cancer Center, 2-3-2, Nakao, Asahi-ku, Yokohama, Kanagawa 241-8515, Japan; Section of Medical Physics and Engineering, Kanagawa Cancer Center, 2-3-2, Nakao, Asahi-ku, Yokohama, Kanagawa 241-8515, Japan; Section of Medical Physics and Engineering, Kanagawa Cancer Center, 2-3-2, Nakao, Asahi-ku, Yokohama, Kanagawa 241-8515, Japan; Department of Radiation Oncology, Kanagawa Cancer Center, 2-3-2, Nakao, Asahi-ku, Yokohama, Kanagawa 241-8515, Japan; Department of Radiation Oncology, Kanagawa Cancer Center, 2-3-2, Nakao, Asahi-ku, Yokohama, Kanagawa 241-8515, Japan; Department of Radiation Oncology, Kanagawa Cancer Center, 2-3-2, Nakao, Asahi-ku, Yokohama, Kanagawa 241-8515, Japan; Department of Radiation Oncology, Kanagawa Cancer Center, 2-3-2, Nakao, Asahi-ku, Yokohama, Kanagawa 241-8515, Japan; Department of Radiation Oncology, Kanagawa Cancer Center, 2-3-2, Nakao, Asahi-ku, Yokohama, Kanagawa 241-8515, Japan; Section of Medical Physics and Engineering, Kanagawa Cancer Center, 2-3-2, Nakao, Asahi-ku, Yokohama, Kanagawa 241-8515, Japan; Department of Gynecology, Kanagawa Cancer Center, 2-3-2, Nakao, Asahi-ku, Yokohama, Kanagawa 241-8515, Japan; Department of Radiation Oncology, Kanagawa Cancer Center, 2-3-2, Nakao, Asahi-ku, Yokohama, Kanagawa 241-8515, Japan

**Keywords:** uterine cervical cancer, adenocarcinoma, scanning carbon-ion radiotherapy, image-guided brachytherapy, cisplatin, concurrent chemoradiotherapy

## Abstract

Although there is growing evidence of the efficacy of carbon-ion radiotherapy (CIRT) for locally advanced cervical adenocarcinoma, reports on combined treatment with CIRT and image-guided brachytherapy (IGBT) are scarce. We retrospectively analyzed patients with International Federation of Gynecology and Obstetrics (2008) stage II–IVA locally advanced cervical adenocarcinoma who received combined scanning CIRT (sCIRT) and IGBT between April 2019 and March 2022. sCIRT consisted of whole-pelvic irradiation with 36 Gy (relative biological effectiveness [RBE]) in 12 fractions and subsequent local boost irradiation with 19.2 Gy (RBE) in 4 fractions. Three sessions of IGBT were administered after completion of sCIRT. Concurrent chemotherapy using weekly cisplatin (40 mg/m^2^/week) was also administered. Efficacy, toxicity and dose–volume parameters were analyzed. Fifteen patients were included in the analysis. The median follow-up period was 25 months. The 2-year overall survival, progression-free survival and local control rates were 92.3% (95% confidence interval [CI] = 77.8–100%), 52.5% (95% CI = 26.9–78.1%) and 84.8% (95% CI = 65.2–100%), respectively. Neither severe acute toxicity necessitating treatment cessation nor grade 3 or higher late toxicity were observed. The sigmoid D_2cm3_ of the patient who developed grade 2 late sigmoid hemorrhage was 65.6 Gy, which exceeded the standard deviation and target dose. The combination of sCIRT and IGBT for locally advanced cervical adenocarcinoma showed acceptable efficacy and safety. Further large-scale and long-term studies are warranted to confirm the efficacy and safety of this treatment.

## INTRODUCTION

Cervical cancer is the fourth most common cancer in women globally, causing 604 000 new cases and 341 000 deaths annually. It is a major cause of death, particularly among young women [1]. Although the widespread use of human papillomavirus vaccines is expected to reduce the risk of invasive cervical cancer, this could take some time in many countries [2]. The radical treatment for cervical cancer includes surgery and (chemo)radiotherapy. For locally advanced stage IIB or higher cervical cancer, concurrent chemoradiotherapy (CCRT) has produced good clinical outcomes, making it the standard of care globally [3].

Based on the histologic types of cervical cancer, although squamous cell carcinoma is the most common type, the incidence of adenocarcinoma increased from ~5% in the 1950s and 1960s to 25% in recent years [4]. CCRT for locally advanced cervical adenocarcinoma has proven inferior in squamous cell carcinoma regarding both overall survival (OS) and local control (LC), and further improvement of the treatment strategy is desired [5].

Carbon-ion radiotherapy (CIRT) is expected to be effective in treating malignant tumors resistant to conventional photon RT [6]. In fact, CIRT has produced good clinical outcomes for several malignant tumors resistant to conventional photon RT [7]. For locally advanced cervical adenocarcinoma, two phase I/II studies of CIRT with/without concurrent cisplatin demonstrated its efficacy [8,9]. Good clinical outcomes of CIRT for locally advanced cervical adenocarcinoma were also reported in a retrospective report from two institutions [10].

Recently, the use of image-guided brachytherapy (IGBT) for cervical cancer has spread globally after the first publication of the Groupe Européen de Curiethérapie European Society for Radiation Oncology (GEC-ESTRO) working group recommendation [11,12]. IGBT makes it possible to deliver adequate radiation doses to large or irregular tumors, resulting in favorable LC rates exceeding 90% regardless of the T stage [13].

Our institution has performed CIRT using the raster scanning method (scanning CIRT [sCIRT]) from 2015 [14], and computed tomography (CT)-based IGBT was also initiated that year. We have additionally treated locally advanced cervical adenocarcinoma using the combination of sCIRT and IGBT since 2019. Historically, when CIRT for cervical cancer was first initiated, brachytherapy (BT) was not utilized. One reason for this is that IGBT had not been developed at the beginning of CIRT, and therefore the treatment accuracy and dosimetry were not adequate. However, another problem was that the CIRT alone caused severe late intestinal toxicity in some cases. In the post-IGBT era, the strategy of replacing part of the local boost of CIRT with IGBT was adopted to optimize treatment in phase I study of Ohno et al., which is the only detailed literature report of combined CIRT and IGBT for locally advanced cervical cancer to date [15]. Because reports on this combination are scarce, data regarding clinical outcomes and radiation dose constraints are desired. Furthermore, compared with passive CIRT, sCIRT could theoretically form a dose distribution that follows the shape of the target. In fact, a comparison of dose distributions in pancreatic cancer CIRT reported that sCIRT are superior to passive CIRT [16]. This study is the first report using combination of sCIRT and IGBT, although Ohno et al. used passive CIRT and IGBT [15].

In this study, we reported the clinical outcomes and dose–volume parameters of the combination of sCIRT and IGBT with concurrent chemotherapy for locally advanced cervical adenocarcinoma at our institution.

## MATERIALS AND METHODS

### Patient eligibility

The database of our institution was reviewed and retrospectively analyzed patients with locally cervical adenocarcinoma who received sCIRT and IGBT between April 2019 and March 2022. The eligibility criteria for this study were as follows: (i) histologically confirmed adenocarcinoma or adenosquamous carcinoma of the uterine cervix, (ii) International Federation of Gynecology and Obstetrics (FIGO, 2008) stage II–IVA, excluding intestinal invasion, (iii) no para-aortic lymph node (PAN) metastases or distant metastases after evaluation by chest-to-pelvis CT, pelvis magnetic resonance imaging (MRI), and whole-body 18F-fluorodeoxyglucose positron emission tomography CT before treatment and (iv) no prior treatment for locally advanced cervical adenocarcinoma.

In all cases, we discussed the indications for treatment at an institutional cancer board consisting of gynecologic oncologists and radiation oncologists. The standard treatment including surgery (if indicated) and photon RT was explained to the patients, and sCIRT was performed in the selected cases.

This study was approved by the institutional review board (approval number: 2022-158), and written informed consent was obtained from all patients.

### Carbon-ion radiotherapy

**Fig. 1:**
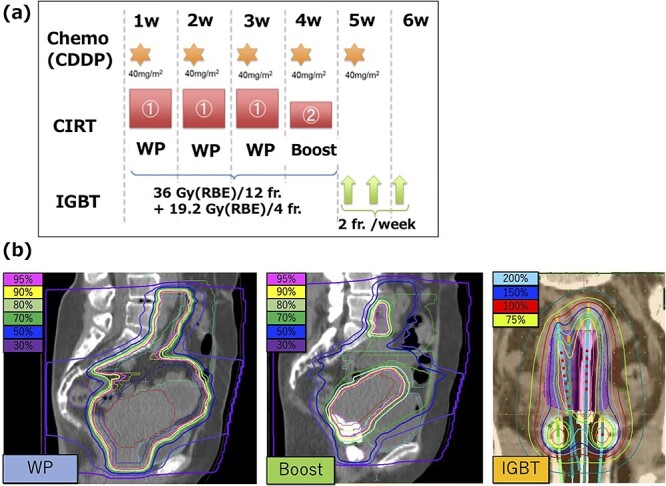
(**a**) Overview of the treatment schedule involving chemotherapy using weekly cisplatin (CDDP), sCIRT and IGBT. (**b**) Representative dose distribution for whole-pelvis sCIRT, boost sCIRT and IGBT. RBE = relative biological effectiveness, fr. = fraction, w = week.

An overview of the treatment schedule, including chemotherapy and the representative dose distributions, is shown in Fig. 1.

CT for treatment planning was performed using patient immobilization devices (underneath: Blue BAG BodyFix [Elekta, Stockholm, Sweden] and upper surface: Shell-fitter [Kuraray, Tokyo, Japan]), and CT images of the maximum exhalation phase (2-mm slices) were taken using treatment planning CT scanner (Aquilion LB [Canon Medical Systems, Tochigi, Japan]). Contouring of the target and organs at risk (OARs) was performed using MIM Maestro version 5.6 (MIM Software Inc, Cleveland, OH, USA). Dose calculation and optimization were performed using Monaco for carbon version 5.20 (Elekta).

sCIRT consisted of whole-pelvic irradiation followed by local boost irradiation (Fig. 1). Radiation doses of sCIRT were expressed in Gy (relative biological effectiveness [RBE]), and irradiation was performed four times a week. The modified microdosimetric kinetic model (mMKM) was used for generating the Gy (RBE) distribution [17].

The gross tumor volumes of the primary tumor (GTVp) and metastatic lymph node (GTVn) were defined by gynecologic examination and imaging modalities. The clinical target volume (CTV) of whole-pelvic irradiation (CTV1) consisted of CTVp, CTVn and CTVsub. CTVp consisted of GTVp plus the uterine cervix, uterine corpus, parametrium, ovaries and at least the upper half of the vagina [18]. CTVp delineated both immediately after urination and after a 100-ml bladder infusion were combined to create ITVp, which was included in CTV1. CTVn was created by adding a 0–5-mm margin to GTVn. CTVsub consisted of the common iliac, internal iliac, external iliac, obturator and presacral lymph nodes [19]. PTV1 was created by adding a 3–5-mm margin to CTV1. Whole-pelvic irradiation was delivered to PTV1 at a prescribed dose of 36 Gy (RBE) in 12 fractions. In whole-pelvic irradiation, one portal was irradiated per day, alternating between the irradiation from the ventral side in the supine position and from the posterior side in the prone position, 6 fractions for each, for a total of 12 fractions. Local boost irradiation was performed by targeting the primary tumor and metastatic lymph nodes. Local boost irradiation of sCIRT was performed prior to IGBT to maximize tumor reduction prior to IGBT. Planning CT images were reacquired before boost irradiation, packing gauzes into the vagina to fix the tumor position and to displace the rectum from the tumor. The CTV for boost irradiation (CTV2) consisted of GTVp plus a 5–10-mm margin, high-risk CTV (CTV_HR_) and CTVn [11,12,20]. PTV2 was created by adding a 0–5-mm margin to CTV2, and the prescribed dose was 19.2 Gy (RBE) in four fractions. In local boost irradiation, one portal was irradiated per day, alternating between the irradiation from the ventral side and lateral side in the supine position, two fractions for each, for a total of four sessions. The number of fractions of each beam direction were changed as necessary, considering location of target lesion and OARs. The target dose of boost irradiation for the rectum and sigmoid D_2cm3_ (the minimum dose delivered to the highest irradiated 2 cm^3^ area) was 4 Gy (RBE) or less, and tumor dose reductions were permitted as necessary.

The verification of the dose distribution in the sCIRT sessions was performed by in-room CT scanner which was set in the treatment room and enabled us to obtain CT images similar to the actual treatment position. In-room CT scanner is the same as the treatment planning CT scanner [21]. In-room CT scan was performed at least once a week to confirm the reproducibility of the dose distribution.

### Image-guided brachytherapy

IGBT was performed twice a week for three sessions after completing sCIRT using an Ir-192 remote after-loading system (microSelectron [Elekta]). Intracavitary brachytherapy (ICBT) was performed using Fletcher and cylindrical applicators. A combination of intracavitary and interstitial BT, the so-called ‘hybrid’ brachytherapy (HBT) was performed in cases in which the physician deemed it necessary for achieving an adequate dose distribution. After the applicators were inserted, images were obtained by CT in the treatment room, and contouring was performed on MIM Maestro with most recent MRI before the first IGBT session (within 1 week) as appropriate. Contouring was based on GEC-ESTRO recommendations and Japanese guidelines [11,12,20]. Treatment planning was performed using Oncentra Brachy version 4.6 (Elekta). The standard treatment planning was performed based on the dose prescription of 5.5 Gy on point A using standard source loading patterns and source dwell weights [22]. The treatment plan was optimized by modifying source positions and dwell times manually on CT images until the treatment plan meets our dose constraints. It is unclear whether fractionated irradiation using the linear-quadratic (LQ) model can be adapted to CIRT [23]. In this study, CIRT dose was evaluated in terms of absolute dose. The target accumulated dose of absolute dose of sCIRT and biological equivalent dose of 2 Gy per fraction (EQD2) of IGBT was <64.1 Gy for rectum and sigmoid D_2cm3_, which is equivalent to accumulation of 40Gy (RBE) in sCIRT and 5 Gy per fraction in IGBT. The target accumulated dose of CTV_HR_ D_90%_ (the minimum dose delivered to 90% of the CTV_HR_) was 76.5 Gy or higher, which is equivalent to accumulation of each prescribed dose of 55.2Gy (RBE) in sCIRT and 5.5 Gy per fraction in IGBT. To ensure safety, dose constraints on the rectum and sigmoid were prioritized and doses to CTV_HR_ were administered as possible.

### Chemotherapy

Considering age and organ function, cisplatin 40 mg/m^2^ was administered once weekly in patients who could tolerate it. Blood tests were performed at least once a week, and the cisplatin dosage was reduced or suspended as appropriate at the discretion of the physician based on myelosuppression, renal function, performance status and other variables.

### Data analysis

OS, progression-free survival (PFS) and LC were calculated using the Kaplan–Meier method. Local relapse was defined as growth or reappearance of the primary tumor in the uterus, parametrium or vagina within the area or at the margins where full radiation dose including whole-pelvic irradiation with both local boost of sCIRT and IGBT were delivered.

Toxicities were evaluated using the National Cancer Institute Common Terminology Criteria for Adverse Events, version 4.0 and classified as acute when they appeared fewer than 3 months after the start of sCIRT and as late otherwise.

Dose–volume parameters were calculated for CTV_HR_ and OARs (bladder, sigmoid and rectum). In sCIRT, the parameters were calculated by summing the parameters for whole-pelvic irradiation and boost irradiation. In IGBT, the parameters were calculated by summing the parameters for each IGBT session. The IGBT doses were converted to EQD2 using a linear quadratic model with an alpha/beta ratio of 3 Gy for the OARs and 10 Gy for CTV_HR_. In sCIRT, dose constraints were set using absolute dose, but both absolute dose and EQD2 were described in the data. The CTV_HR_ D90% was compared between patients with and without local relapse using t-test. As supplementary analysis for verifying the difference of irradiated dose between the treatment plan and the actual treatment session, we analyzed the dose–volume parameters of the sigmoid of the patient who developed grade 2 sigmoid hemorrhage using in-room CT images and deformable image registration (DIR), which takes into account the interfractional displacement and deformation of organ. The DIR was performed by combining image information by intensity with the contoured structure of the sigmoid using MIM Maestro version 5.6 (MIM Software Inc, Cleveland, OH, USA). The CIRT dose distribution of the in-room CT and IGBT dose distribution in CT were deformed by DIR and integrated with the CT of the first session of IGBT to evaluate the sigmoid dose. We used IBM SPSS Statistics software, version 26.0 (IBM, Armonk, NY, USA) for all statistical analyses.

## RESULTS

### Patient and treatment characteristics

Fifteen patients with locally advanced cervical adenocarcinoma initiated CIRT between April 2019 and March 2022 and met the eligibility criteria. Patient characteristics are summarized in Table 1. The median age and follow-up period were 61 years and 25 months (range, 9–42), respectively. According to the Union for International Cancer Control TNM Classification of Malignant Tumors, 8th edition, the T stage was 2b in 11 patients (73%) and 3b in 4 patients (27%). The N stage was 0 in 4 patients (27%) and 1 in 11 patients (73%). The FIGO (2018) stages were IIB, IIIB and IIIC1r in 3 (20%), 1 (6.7%) and 11 patients (73%), respectively. The median maximum diameter of the primary tumor was 5.5 cm (range, 3.3–6.6). The histology was adenocarcinoma in 12 patients (80%) and adenosquamous carcinoma in 3 patients (20%). The median number of cisplatin courses was 5 (range, 3–6), and the median total administered dose was 200 mg (range, 90–240). Meanwhile, 11 patients (73%) received ICBT, and 4 (27%) received HBT (at least once). The T stage of the 4 patients who underwent HBT was T2b in 2 patients and 3b in 2 patients. The median overall treatment time (OTT) was 37 days (range, 35–42).

**Table 1:** Patient and treatment characteristics

Patient characteristics	*n* = 15
Age at diagnosis (median), years	45–83 (61)
Follow-up period (median), months	6–42 (24)
T stage, *n* (%)	
2b	11 (73)
3b	4 (27)
N stage, *n* (%)	
0	4 (27)
1	11 (73)
FIGO stage	
IIB	3 (20)
IIIB	1 (6.7)
IIIC1r	11 (73)
Tumor diameter (median), cm	3.3–6.6 (5.5)
Histology, *n* (%)	
Adenocarcinoma	12 (80)
Adenosquamous carcinoma	3 (20)
Treatment characteristics	
Weekly CDDP	
Yes, *n* (%)	15 (100)
Cycles (median), *n*	3–6 (5)
Total dose (median), mg/m^2^	90–240 (200)
BT method, *n* (%)	
ICBT	11 (73)
HBT	4 (27)
OTT (median), days	35–42 (37)

Abbreviations: FIGO = International Federation of Gynecology and Obstetrics, CDDP = cisplatin, ICBT = intracavitary brachytherapy, HBT = hybrid intracavitary and interstitial brachytherapy, OTT = overall treatment time.

### Efficacy

**Fig. 2:**
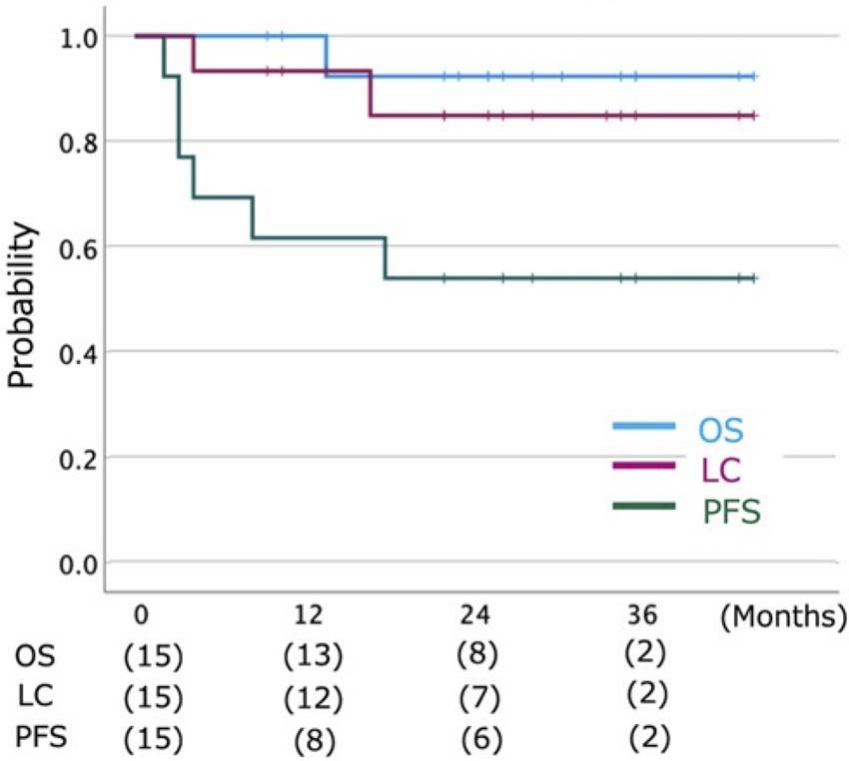
Kaplan–Meier curves of OS, (LC and PFS for all 15 patients. The number at risk is shown below the figure.

The 2-year OS, PFS and LC rates were 92.3% (95% confidence interval [CI] = 77.8–100%), 52.5% (95% CI = 26.9–78.1%) and 84.8% (95% CI = 65.2–100%), respectively (Fig. 2).

The patterns of the relapse are summarized in Table 2. Of the seven first relapses, one occurred within the irradiated field, four occurred outside the irradiated field and two occurred in both. Five first relapses included PAN metastasis. Three patients developed PAN relapse alone and received salvage local treatment with photon RT and CIRT. Two patients with relapse involving PANs and other distant sites received platinum-doublet chemotherapy.

**Table 2:** Summary of the patterns of relapse

Site of first relapse	Time to relapse (months)	Treatment after relapse	Local relapse	TNM stage	Initial metastatic LN
PAN	17	CIRT	No	T2bN1M0	Obturator
Primary lesion	4	Chemotherapy (TC)	Yes	T3bN0M0	None
PAN	2	Photon RT	No	T2bN1M0	Obturator, common iliac
Pelvic LN, PAN,					
mediastinal LN, axillary LN	8	Chemotherapy (TC)	Yes	T2bN1M0	Obturator
PAN	3	CIRT	No	T2bN1M0	Obturator, common iliac
Peritoneal metastasis	3	Chemotherapy (TC)	No	T2bN0M0	None
PAN, supraclavicular LN, ovaries	3	Chemotherapy (TP)	No	T3bN1M0	Obturator

Abbreviations: PAN = para-aortic lymph node, LN = lymph node, CIRT = carbon-ion radiotherapy, RT = radiotherapy, TC = carboplatin and paclitaxel, TP = cisplatin and paclitaxel.

Two patients experienced local relapse during follow-up (Table 2). Of those two patients, T stage was 2b in one patient and 3b in the other patient. The T2b patient received ICBT and T3b patient received HBT. In both of these two patients, re-enlargement of the primary cervical tumor that once shrunk was observed. One lesion was unresectable at the time of relapse, and one patient had a prior distant relapse. Therefore, salvage surgery was not performed, and platinum-doublet chemotherapy was administered. One patient had a recurrence in the ovary, but it was not considered a local relapse because there was no lesion in the ovary prior to treatment and it was a metastatic recurrence in a site within the sCIRT whole-pelvis irradiation field, but without sCIRT local boost or IGBT doses.

### Toxicities

Acute and late toxicities are summarized in Table 3. Concerning acute hematologic toxicities, one patient each (6.7%) developed grade 3 neutropenia and grade 3 anemia. Grade 3 liver injury occurred in one patient (6.7%), leading to the suspension of chemotherapy. Regarding acute non-hematologic toxicity, one patient developed grade 3 uterine infection (6.7%), which occurred 2 months after treatment initiation and 1 month after treatment completion. The infection improved immediately after drainage of the pyometra and intravenous antibiotics administration. Meanwhile, grade 2 diarrhea developed in three patients (20%). sCIRT and IGBT were completed without interruption in all patients.

**Table 3:** Acute and late toxicities

Acute (*n* = 15)	G0	G1	G2	G3
Neutropenia	8	4	2	1
Anemia	2	7	5	1
Thrombocytopenia	12	3	0	0
AST/ALT elevation	8	6	0	1
Creatinine elevation	11	4	0	0
Nausea	5	10	0	0
Diarrhea	7	5	3	0
Uterine infection	0	0	0	1
				
Late (*n* = 15)	G0	G1	G2	≥G3
GU	12	1	2^a^	0
GI	14	0	1	0
Bone	12	2	1	0

^a^One patient developed bladder fistula attributable to relapse of the primary tumor.

Abbreviations: AST = aspartate aminotransferase, ALT = alanine aminotransferase, G = grade.

No grade 3 or higher late toxicity was observed. Grade 2 late toxicities were observed in four patients (27%), including sigmoid hemorrhage, vesicovaginal fistula (associated with relapse of the primary tumor) and noninfectious cystitis in one patient each. Grade 2 lumbar vertebral compression fracture (in the irradiated field) was also observed in one patient. Sigmoid bleeding occurred 9 months after CIRT initiation, and the event was improved by laxatives and salazopyrine enema. Noninfectious cystitis with bleeding and pain developed 15 months after CIRT initiation, and this event was relieved by hyperbaric oxygen therapy.

### Dose–volume parameters

Dose–volume parameters for the entire patient population are summarized in Table 4. In sCIRT, dose constraints were set using absolute dose, but both absolute dose and EQD2 were described in the data. The mean ± standard deviation (SD) of CTV_HR_ D_90%_ for combined absolute dose of sCIRT and EQD2 of IGBT and EQD2 of sCIRT and EQD2 of IGBT were 81.8 ± 2.63 Gy and 88.2 ± 3.08 Gy, respectively. The target dose of CTV_HR_ D_90%_ (absolute dose of sCIRT and EQD2 of IGBT ≥76.5 Gy) was met in all patients. The mean ± SD of rectum D_2cm3_ and sigmoid D_2cm3_ for combined absolute dose of sCIRT and EQD2 of IGBT were 57.4 ± 3.76 Gy and 54.0 ± 7.73 Gy. The target dose of rectum D_2cm3_ and sigmoid D_2cm3_ (absolute dose of sCIRT and EQD2 of IGBT <64.1 Gy) was met in all patients for the rectum and 13 patients (87%) for the sigmoid.

**Table 4:** Dose–volume parameters of CIRT and IGBT

	CIRT (absolute) (Gy [RBE])	CIRT (EQD2) (Gy [RBE])	IGBT EQD2 (Gy)	CIRT (absolute) + IGBT (Gy, Gy[RBE])	CIRT (EQD2) + IGBT (Gy, Gy[RBE])
CTV_HR_ D_90%_	53.2 ± 1.79	59.7 ± 2.70	28.6 ± 2.61	81.8 ± 2.63	88.2 ± 3.08
CTV_HR_ D_98%_	49.3 ± 3.22	54.1 ± 4.58	22.6 ± 2.73	71.9 ± 3.41	76.7 ± 4.46
Bladder D_2cm3_	53.2 ± 3.35	68.7 ± 6.28	28.2 ± 4.76	81.4 ± 7.04	96.9 ± 9.62
Rectum D_2cm3_	39.6 ± 1.66	46.2 ± 1.76	17.8 ± 3.14	57.4 ± 3.76	64.0 ± 3.81
Sigmoid D_2cm3_	40.0 ± 3.10	46.9 ± 3.30	14.0 ± 6.06	54.0 ± 7.73	61.0 ± 7.96

Abbreviations: RBE = relative biological effectiveness, EQD2 = a biological equivalent dose of 2 Gy per fraction, CTV_HR_ = high-risk clinical target volume, CIRT = carbon-ion radiotherapy, IGBT = image-guided brachytherapy.

The mean of CTV_HR_ D_90%_ for combined absolute dose of sCIRT and EQD2 of IGBT was not significantly different between patients with and without local relapse (81.3 vs. 81.9 Gy, *P* = 0.490). The mean of CTV_HR_ D_90%_ for combined EQD2 of sCIRT and EQD2 of IGBT was also not significantly different between patients with and without local relapse (87.0 vs. 88.4 Gy, *P* = 0.167). The absolute dose of sCIRT and EQD2 of IGBT bladder D_2cm3_ for the patient with grade 2 noninfectious cystitis was 80.4 Gy, which was within the SD range. The absolute dose of sCIRT and EQD2 of IGBT sigmoid D_2cm3_ for the patient with grade 2 sigmoid hemorrhage was 65.6 Gy, which exceeded the SD range and target dose.

## DISCUSSION

In this study, we reported the clinical outcomes and dose–volume parameters of combined sCIRT and IGBT for locally advanced cervical adenocarcinoma. This combined treatment has been reported only in one phase I study and part of one retrospective study [10,15]. Therefore, data on this treatment are still insufficient.

In photon RT, the recent development of IGBT has allowed for improved outcomes as definitive treatment [13]. However, despite the development of IGBT, the outcomes of adenocarcinoma remain poor compared with squamous cell carcinoma [5,24,25]. CIRT was introduced to enhance outcomes in radioresistant cervical adenocarcinoma.

Previous reports of photon RT and CIRT for cervical adenocarcinoma and the results of this study are summarized in Table 5 [8–10,26–29]. In studies by Wakatsuki et al. [8] using CIRT alone and Okonogi et al. [9] using CIRT with weekly cisplatin, CIRT was administered without IGBT. Meanwhile, in a separate study by Okonogi et al., 65% of patients received concurrent chemotherapy, and 11% received combined CIRT and IGBT [10]. These reports revealed 2- and 5-year OS of 66–87% and 38–69% and 2- and 5-year LC of 60–74% and 55–65%, respectively. They also indicated improved OS and LC with combined chemotherapy compared with CIRT alone [8–10]. Conversely, all patients in the present study received concurrent chemotherapy and IGBT. In this study, 2-year OS was 92% and LC 85%. Although our follow-up period was relatively short, the results of this study are comparable to those of previous reports of CIRT, especially in combination with chemotherapy. In this study, there were trends toward a slightly lower T3 rate and a higher N1 rate versus previous reports, and these trends should be carefully monitored for their impact on treatment outcomes. For photon RT, four retrospective studies reported 2- and 5-year OS of 52–70% and 41–50%, respectively, as well as 5-year PFS of 30–36%, and 5-year LC of 62%–64% [26–29]. The more recent OUTBACK trial, which investigated the superiority of adding adjuvant chemotherapy to CCRT, reported a 71% 5-year OS and 53% PFS in the adenocarcinoma subgroup in the CCRT alone arm, with these rates surpassing those reported in previous retrospective studies [30]. Although the results in the present study are superior to those in the four aforementioned retrospective studies, it remains imperative to confirm the long-term outcomes of CIRT + IGBT and compare them with the results of adenocarcinoma in the most recent photon RT clinical trials.

**Table 5:** Summary of reports on Photon RT and CIRT for cervical adenocarcinoma

Author (Year)	Study design	No. of patients	EBRT	BT	CT	Median follow-up (months)	T stage (≥T3)	N stage (N1)	OS		PFS		LC		≥G3 late toxicity
									2 y	5 y	2 y	5 y	2 y	5 y	
Chen et al. (2014)	Multi-institutional retrospective	35	Photon	Yes (93%)	Yes (60%)	59	2b, 3, 4a (31%)	N/R	73%	41%	N/R	30%	N/R	64%	23%
Zhang et al. (2020)	Multi-institutional retrospective	331	Photon	Yes (79%)	Yes (N/R)	41	1b, 2, 3, 4a (34%)	N/R	52% (3 y)	43%	N/R	N/R	N/R	N/R	N/R
Miyasaka et al. (2020)	Multi-institutional retrospective	71	Photon	Yes (100%)	Yes (60%)	37	1b, 2, 3, 4a (49%)	0,1 (45%)	70%	50%	N/R	36%	N/R	62%	N/R
Okonogi et al. (2022)	Multi-institutional retrospective	36	Photon	Yes (100%)	Yes (81%)	39	1b, 2, 3, 4a (N/R)	0,1 (56%)	68% (3 y)	N/R	44% (3 y)	N/R	69% (3 y)	N/R	5.6%
Mileshikin et al. (2023)	Multi-institutional prospective	79	Photon	Yes (N/R)	Yes (N/R)	60	1b, 2, 3, 4a (N/R)	0, 1 (N/R)	N/R	71%	N/R	53%	N/R	N/R	N/R
Wakatsuki et al. (2014)	Single-institutional prospective	55	CIRT	No	No	38	2b, 3b, 4a (64%)	0,1 (44%)	66%	38%	N/R	N/R	60%	55%	GU 0% GI 3.4%
Okonogi et al. (2018)	Single-institutional prospective	31	CIRT	No	Yes (100%)	30	2b, 3b, 4a (39%)	0,1 (39%)	84%	N/R	56%	N/R	74%	N/R	GU 0% GI 6.4%
Okonogi et al. (2021)	Multi-institutional retrospective	55	CIRT	Yes (11%)	Yes (65%)	67	2b, 3b, 4a (33%)	0,1 (40%)	87%	69%	N/R	44%	N/R	65%	GU 5.4% GI 9.1%
Present study	Single-institutional retrospective	15	CIRT	Yes (100%)	Yes (100%)	25	2b, 3b (27%)	0,1 (73%)	92%	N/R	54%	N/R	85%	N/R	GU 0% GI 0%

Abbreviations: CIRT = carbon-ion radiotherapy, BT = brachytherapy, CT = chemotherapy, OS = overall survival, PFS = progression-free survival, LC = local control, y = years, NR = not reported, G = grade, GU = genitourinary, GI = gastrointestinal.

With regard to the pattern of recurrence in this study, the majority of recurrences included PAN in N1 patients. Although there are strategies for salvage RT for solitary relapses, the outcome after PAN relapse is not sufficient [31]. Therefore, it is important for N1 patients to prevent PAN relapse to improve treatment outcomes. To prevent PAN recurrence, strategies of both RT and chemotherapy needs to be reviewed. First, with regard to RT strategy, one approach is to reconsider the irradiation field, since all PAN recurrences in this study were outside the whole-pelvic irradiation field. The EMBRACE II study, which aims to optimize definitive RT methods for cervical cancer, is using risk stratification according to the pelvic lymph node status, and prophylactic irradiation of the PAN region is being applied in high-risk pelvic nodes positive patients [32]. Wakatsuki et al. reported the results of a phase I/II study of extended-field CIRT including the PAN region for locally advanced cervical squamous cell carcinoma. In the study, the PAN relapse rate was 5.3%, which was lower than that of conventional pelvic field CIRT, and no grade 3 or higher acute or late toxicities were identified [33]. Because the study examined data from squamous cell carcinoma, it is unclear whether an extended-field is also effective in CIRT for adenocarcinoma. However, prophylactic irradiation of the PAN region may be effective strategy. Another approach is to prevent PAN recurrence by increasing the intensity of chemotherapy. Recently, positive results have been obtained from the INTERLACE trial, which added neoadjuvant chemotherapy to photon CCRT for locally advanced cervical cancer, and the KEYNOTE-A18 trial, which added immune checkpoint inhibitor as concurrent and adjuvant treatment [34,35]. Adapting these chemotherapy efforts to CIRT may contribute to the prevention of PAN and other irradiated field recurrences, and future treatment development is encouraged. Actually, the protocol for a phase Ib study to add an immune checkpoint inhibitor to CIRT to improve treatment intensity has been reported [36].

Furthermore, two local relapses occurred in the form of the re-enlargement of the primary cervical tumor that once shrunk. A reduction of local relapses may also be achieved with improved RT and chemotherapy strategies. In photon RT, higher dose is required for LC in adenocarcinoma compared with squamous cell carcinoma, and the increasing the accumulated dose of sCIRT and IGBT is the first countermeasure [5]. The aggressive use of HBT on IGBT and the use of gel spacers are techniques that increase the target dose without increasing the gastrointestinal (GI) dose [37,38]. In addition, the linear energy transfer (LET) distribution has been reported to affect LC. Hagiwara et al. found that LET to GTV was associated with LC in locally advanced pancreatic cancer [39]. In an *in vitro* study, Shiba et al. reported a difference in survival rate with varying LET in CIRT of HeLa cells in human cervical adenocarcinoma [40]. Furthermore, the passive and scanning methods may result in slight differences in LET due to the different methods of forming the dose distribution. It should be noted that although they are the same CIRT and IGBT, the phase I study by Ohno et al. uses a passive method, while this study uses a scanning method [15]. Because this study could not obtain the on LET, further investigation into LET as a factor related to LC is warranted.

Regarding acute toxicity, Okonogi et al. reported one case of grade 3 or higher hematologic toxicity and one case of grade 3 or higher non-hematologic toxicity among 22 patients in a phase I/II study of CIRT with concurrent chemotherapy [9]. In a phase I study of combined CIRT and IGBT (six patients), Ohno et al. reported one case of grade 3 neutropenia, and no patient developed grade 2 or higher non-hematologic toxicity [15]. The pattern of adverse events was similar in this study, and it was possible to complete the planned RT without interruption.

In previous reports of CIRT, the rates of grade 3 or higher late genitourinary (GU) and GI toxicities, including those attributable to tumor relapse, ranged 0–5.4% and 3.4–9.1%, respectively [8–10]. Ohno et al. observed one case of grade 3 rectal bleeding among six patients in a phase I study of combined CIRT and IGBT [15]. Regarding photon RT, Pötter et al. reported that the 5-year rates of grade 3–5 GU and GI toxicities were 6.8 and 8.5%, respectively, in a large multicenter cohort study using MRI-based IGBT [13]. In a Japanese phase I/II study of HBT, Murakami et al. noted that 3.8% of patients developed grade 3 or higher GI toxicity [25]. From prior results and those of the present study, the combination of CIRT and IGBT is considered safe. However, because of the relatively short follow-up period, caution should be exercised regarding the possibility of increased toxicity, especially bladder and small intestine toxicity, in the future [41]. Insufficiency fracture is also an important factor that lowers the quality of life, and it has been reported following CIRT [42,43]. It is desirable to devise irradiation methods to prevent insufficiency fractures.

Concerning dose–volume parameters, there are no well-established indicators for combined CIRT and IGBT. Regarding photon RT, the EMBRACE study identified the benchmark dose constraints [13]. As a dose factor predicting toxicities in CIRT for cervical cancer, Kato et al. reported that the GI dose should be <60 Gy (RBE) to prevent major late GI complications [44]. More recently, Okonogi et al. demonstrated via a univariate analysis of pooled data of patients with cervical cancer that rectum D_2cm3_ was a significant predictor of >grade 1 late proctitis and bladder D_5cm3_ was predictive of >grade 1 late cystitis [45]. However, both reports investigated CIRT alone (without IGBT), making direct comparison with the present study difficult. Ohno et al. reported parameters including CTV_HR_ D_90%_, bladder D_2cm3_ and rectosigmoid D_2cm3_ in treated patients in a phase I study of combined CIRT and IGBT [15]. In the study, EQD2 of CIRT was used for accumulated dose evaluation. On the other hand, in the present study, the dose constraints based on the sum of the absolute dose of sCIRT and the EQD2 of IGBTs were set against the background that the LQ model may not be valid for CIRT [23]. The dose constraints for the rectum and sigmoid in this study were 70.4 Gy when the CIRT portion was converted to EQD2, which was similar to the recommended dose of 67.2–71.3 Gy in the phase I study by Ohno et al. [15]. Because our study size was limited, it is necessary to examine the validity of dose constraints by accumulating more data in the future. In particular, whether absolute or EQD2 dose of CIRT is a better predictor of LC and adverse events is an important question to be addressed in future studies.

Variations in the reproducibility of the dose distribution in actual treatment sessions in CIRT have been reported to be caused by variations in organ position, body shape and other variables [46,47]. However, reports of intra- and inter-fractional errors in CIRT for cervical cancer are scarce. Because critical organ motion during the treatment period has been reported in photon RT for cervical cancer, it is important to consider this issue in CIRT for cervical cancer [48]. In our institution, an initial approach involved using a computer-aided online 2D–3D positioning program with orthogonal kilovoltage X-ray flat panel detector images in each treatment session. This facilitated patient positioning with an accuracy of <1 mm based on bone structures. Immediately before or after irradiation, in-room CT (CT-on-rails) images were captured under the treatment conditions during sessions occurring at least once per week. Then, dose verification in an actual treatment session is performed. As pilot data, we analyzed the dose–volume parameters of the patient who developed grade 2 sigmoid hemorrhage using in-room CT and DIR, which takes into account the displacement and deformation of organs. In the analysis, the dose to the sigmoid, which was calculated using in-room CT and DIR, was higher than the initial assumption in planning CT (Fig. S1, Table S1). Thus, it is important to examine the correlation between dose–volume parameters and specific events, considering both the dose at the time of treatment planning and that during actual irradiation to improve treatment outcomes in the future.

The limitations of this study included the relatively small number of patients, the retrospective nature of the data and the short median follow-up period of ~2 years; therefore, the data are not mature. Although we mentioned dose–volume parameters and specific events, there are some variations in the methods of such analyses. In addition, the number of CIRT facilities is limited worldwide, with 14 worldwide and 7 in Japan as of 2023. However, the number of facilities has been increasing recently and there are now ~20 facilities including those under construction [49]. On the other hand, reports of CIRT for cervical cancer have been limited to Japan, and clinical trials aimed at establishing further high-level evidence and global development are desired [8–10,15].

The combination of sCIRT and IGBT for locally advanced cervical adenocarcinoma showed acceptable efficacy and safety. Further large-scale and long-term studies are warranted to confirm the efficacy and safety of this treatment.

## PRESENTATION AT A CONFERENCE

Part of this study was presented at the 35th annual meeting of the Japanese Society for Radiation Oncology, 2022.

## Supplementary Material

SupplementaryFigureS1_JRR_rrae043

SupplementaryTable_rrae043
