# Supplementary material for: Preliminary result of combined treatment with scanning carbon-ion radiotherapy and image-guided brachytherapy for locally advanced cervical adenocarcinoma
Source: J Radiat Res. 2024 Jun 6;65(4):512–22. doi: 10.1093/jrr/rrae043 (PMC11262861; doi:10.1093/jrr/rrae043)
Supplement: SupplementaryFigureS1_JRR_rrae043 [file supplementaryfigures1_jrr_rrae043.docx]

**Fig. S1.** (a) In-room CT images were acquired in the actual treatment position immediately after treatment. (b) Reproduction of the dose distribution of boost irradiation of carbon-ion radiotherapy on in-room CT using DIR. A high radiation dose beyond expectation was irradiated to the sigmoid (pink-colored structure).
