# Supplementary material for: Preliminary result of combined treatment with scanning carbon-ion radiotherapy and image-guided brachytherapy for locally advanced cervical adenocarcinoma
Source: J Radiat Res. 2024 Jun 6;65(4):512–22. doi: 10.1093/jrr/rrae043 (PMC11262861; doi:10.1093/jrr/rrae043)
Supplement: SupplementaryTable_rrae043 [file supplementarytable_rrae043.docx]

| Table S1. Dose–volume parameters of the sigmoid in the patient who developed grade 2 sigmoid hemorrhage | | | | | | |
| --- | --- | --- | --- | --- | --- | --- |
| Planning CT | | | | | | |
| Simple accumulated addition | Initial CIRT | Boost CIRT | IGBT1 (EQD2) | IGBT2 (EQD2) | IGBT3 (EQD2) | Total dose |
| Dmax | 36.19 | 16.03 | 19.99 | 9.18 | 49.44 | 130.84 |
| D0.03cc | 36.09 | 14.40 | 17.31 | 8.57 | 40.34 | 116.70 |
| D0.1cc | 36.02 | 13.35 | 15.60 | 8.03 | 34.36 | 107.36 |
| D1cc | 35.57 | 10.05 | 10.24 | 6.24 | 16.66 | 78.75 |
| D2cc | 34.68 | 8.25 | 8.22 | 5.50 | 11.54 | 68.19 |
| DIR addition |  |  |  |  |  | Total dose |
| Dmax |  |  |  |  |  | 147.89 |
| D0.03cc |  |  |  |  |  | 113.20 |
| D0.1cc |  |  |  |  |  | 104.89 |
| D1cc |  |  |  |  |  | 79.74 |
| D2cc |  |  |  |  |  | 70.72 |
| In-Room CT | | | | | | |
| Simple accumulated addition | Initial CIRT | Boost CIRT | IGBT1 (EQD2) | IGBT2 (EQD2) | IGBT3 (EQD2) | Total dose |
| Dmax | 36.55 | 19.22 | 19.99 | 9.18 | 49.44 | 134.38 |
| D0.03cc | 36.44 | 18.99 | 17.31 | 8.57 | 40.34 | 121.65 |
| D0.1cc | 36.39 | 18.77 | 15.60 | 8.03 | 34.36 | 113.15 |
| D1cc | 36.18 | 17.69 | 10.24 | 6.24 | 16.66 | 87.00 |
| D2cc | 36.04 | 16.58 | 8.22 | 5.50 | 11.54 | 77.88 |
| DIR addition |  |  |  |  |  | Total dose |
| Dmax |  |  |  |  |  | 154.44 |
| D0.03cc |  |  |  |  |  | 120.56 |
| D0.1cc |  |  |  |  |  | 111.04 |
| D1cc |  |  |  |  |  | 83.49 |
| D2cc |  |  |  |  |  | 73.34 |
| Dose of CIRT is presented as Gy (RBE), and IGBT and Total dose is presented as Gy. | | | | |  |  |
| Simple accumulated addition represents doses calculated in each planning or in-room CT without DIR method. | | | | | | |
| DIR addition represents accumulated doses using DIR methods for each CT. | | | |  |  |  |
| Abbreviations: CIRT, carbon-ion radiotherapy; IGBT, image-guided brachytherapy; DIR, deformable image registration; EQD2, equivalent dose in a 2-Gy fraction | | | | | | |
